# Primary Vaginal Mucinous Adenocarcinoma of Intestinal Type—Clinical, Radiological and Morphological Aspects

**DOI:** 10.3390/medicina60040525

**Published:** 2024-03-22

**Authors:** Tatyana Betova, Radoslav Trifonov, Savelina Popovska, Angel Yordanov, Konstantina Karakadieva, Zhivka Dancheva, Stoyan Kostov

**Affiliations:** 1 Department of Pathologoanatomy, Medical University—Pleven, 5800 Pleven, Bulgaria; betova@abv.bg (T.B.); sapopovska@yahoo.com (S.P.); 2 Department of Radiology and Radiotherapy, Medical University—Pleven, 5800 Pleven, Bulgaria; 3 Department of Gynecological Oncology, Medical University—Pleven, 5800 Pleven, Bulgaria; angel.jordanov@gmail.com (A.Y.); kkarakadieva@gmail.com (K.K.); 4 Department of Imaging Diagnostics, Interventional Radiology and Radiotherapy, Medical University—“Prof. Dr. Paraskev Stoyanov”, 9002 Varna, Bulgaria; dr.dancheva@gmail.com; 5 Department of Gynecology, Hospital “Saint Anna”, Medical University—“Prof. Dr. Paraskev Stoyanov”, 9002 Varna, Bulgaria; drstoqn.kostov@gmail.com; 6 Research Institute, Medical University—Pleven, 5800 Pleven, Bulgaria

**Keywords:** vaginal tumors, primary mucinous adenocarcinoma intestinal type, vaginal adenoma, immunohistochemistry, radiology, treatment

## Abstract

*Background and Objectives*: Neoplasms of the vagina are rare and account for 1–2% of all tumors of the female reproductive system. Primary neoplasms of the vagina are most often carcinomas originating from squamous or glandular epithelium. Of the primary glandular tumors, clear cell, endometrioid, and serous adenocarcinomas are the most common types, while mucinous and mesonephric types are very rare. Mucinous adenocarcinoma is histologically subclassified into endocervical and intestinal types. We add to the existing literature another case of an extremely rare gynecological neoplasm—primary vaginal mucinous adenocarcinoma (PVMAC) intestinal type associated with vaginal villous adenoma with high-grade dysplasia. We discuss the clinical, radiological and morphological features of this rare entity. *Materials and Methods*: We report a case of a 59-year-old woman with PVMAC intestinal type associated with vaginal villous adenoma with high-grade dysplasia. The patient was evaluated with a gynecological exam, and biopsy, curettage and tumor excision were performed. The positron emission tomography-computed tomography (PET/CT) scan, at the level of the pelvis, supported the primary location of the disease. Histological and immunohistochemical methods were applied. *Results*: The gynecological examination of the vagina revealed an exophytic polypoid mass with a diameter of 3 cm, located on the posterior wall, in the area of introitus vaginae. The PET/CT scan revealed a hypermetabolic malignant formation involving the vagina and anal canal, without evidence of pelvic and inguinal lymphadenopathy, and also, it excluded disease at sites other than the vagina. The histological and immunohistochemical investigations, as well as the clinical and radiological data, lent support to the diagnosis “primary vaginal mucinous adenocarcinoma intestinal type”. *Conclusions*: PVMAC intestinal type is a rare gynecological pathology, which presents a serious challenge for oncogynecologists, radiologists and pathologists.

## 1. Introduction

Globally, in 2020, there were 19.3 million newly registered oncological patients, of which 16.5% were gynecological oncological diseases. Newly registered malignant diseases in the female population were 8.2 million, which were most often localized in the cervix, endometrium and ovaries [1,2]. Neoplasms of the vagina were very rare and accounted for 1–2% of all tumors of the female reproductive system, and in 91% of cases, they were of metastatic origin [3]. Of the primary vaginal tumors, the most prevalent was squamous cell carcinoma (75%), followed by adenocarcinoma (5–10%) and all other rare variants including malignant melanoma, lymphomas and sarcomas [3,4]. Of the adenocarcinomas, the most frequent were clear cell, endometrioid and serous adenocarcinomas, while mucinous and mesonephric histological variants were very rare [4,5,6]. Mucinous adenocarcinoma is subclassified into endocervical and intestinal types [7]. PVMAC intestinal type is one of the rarest, with 20 cases described in the English literature, of which only nine cases were associated with tubular, villous and tubulo-villous adenomas.

We present a case of PVMAC intestinal type, arising as part of a villous adenoma with high grade dysplasia.

## 2. Case Presentation

A 59-year-old woman was admitted to the Oncology Clinic in October 2022 with complaints of copious mucus vaginal discharge and small amounts of irregular vaginal bleeding in the postmenopausal period. The patient had one birth and had been in menopause for 9 years. She had been sexually inactive for the last 6 years and her gynecological visits were irregular. From the past medical history, there was information about an ischemic brain stroke in 2022, arterial hypertension, endoprosthesis of the right knee joint, nodular goiter and allergies to food and medications. There was no family history of gynecological or any other malignancies.

**Figure 1:**
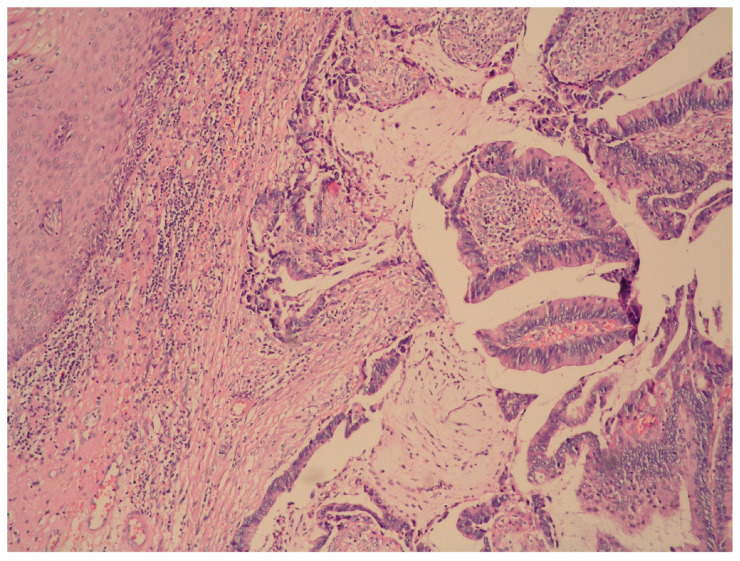
Vaginal wall lined by non-keratinizing squamous epithelium (left upper corner) and area with the characteristic features of intestinal type villous adenoma with high-grade dysplasia; hematoxylin and eosin (H&E) stain, magnification ×400.

From the gynecological examination, the external genitalia were normal. The examination of the vagina revealed to the oncogynecologistabundant discharge of mucus and an exophytic polypoid tumor formation with a diameter of 3 cm, located on the posterior wall, in the area of introitus vaginae. Uterus and adnexa—no features. The result from her Pap smear was negative for malignancy. A digital rectal examination was performed, which established an intact mucosa of the anterior rectal wall without invasion and connection to the vaginal lesion. Regional lymphadenomegaly and infiltration of the paravaginal and rectovaginal spaces were absent. Biopsy and curettage were performed, which revealed an intestinal type villous adenoma with high-grade dysplasia with an inability of evaluating its base (Figure 1). The endometrial biopsy was normal.

**Figure 2:**
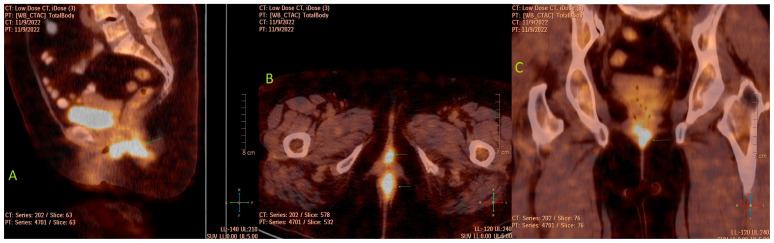
[^18^F] FDG PET/CT scan fused images: (**A**) sagittal view, metabolically active formation in the vagina, reaching the anal canal (green arrow); (**B**) axial view, metabolically active formation in the vagina and a zone of high activity in the anal canal, which might be due to infiltration from the established tumor or inflammatory changes related to hemorrhoidal disease (green arrows); (**C**) coronal view, metabolically active formation in the vagina (green arrow).

The patient also underwent a PET/CT scan, which was required to exclude disease at sites other than the vagina. It revealed a hypermetabolic malignant formation involving the vagina and anal canal with axial dimensions of 54/28 mm, SUVmax10 (Figure 2), without pelvic and inguinal lymphadenopathy nor distant lesions. The differential diagnosis of the high metabolic activity in the anal canal included inflammatory changes related to hemorrhoidal disease. This necessitated rectoscopy, which excluded an infiltrative process of the mucosa.

**Figure 3:**
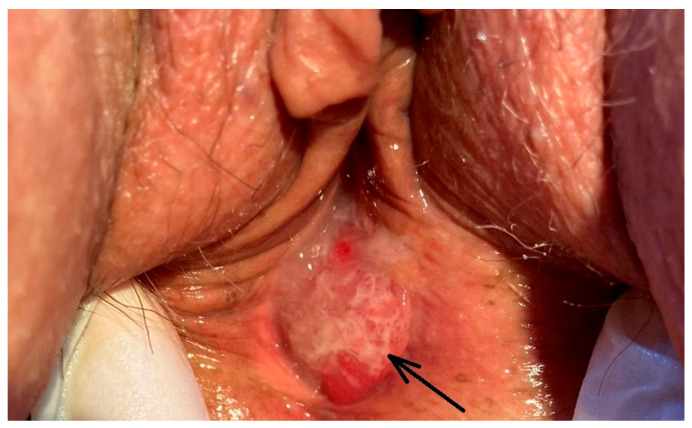
Polypoid tumor formation on the posterior vaginal wall with superficial mucinous secretion (arrow).

One month later, the tumor was removed and a posterior colporrhaphy was performed. Preoperative laboratory tests showed data on anemia: RBC (red blood cells)—2.73 × 10^12^/L (reference range 3.7–5.3 × 10^12^/L); HGB (hemoglobin)—96.0 g/L (reference range 120–160 g/L); hematocrit (HCT)—0.289/L (reference range 0.36–0.48/L), mean corpuscular volume (MCV)—106 ft (reference range 82–96 ft); mean corpuscular hemoglobin (MCH)—35.4 pg (reference range 27–33 pg), while the other indicators, including tumor markers for cervical, ovarian and gastrointestinal neoplasms, were within normal values: CA125—20 U/mL (normal < 35 U/mL) and CA19-9—25 U/mL (normal < 35 U/mL), apart from CEA, which was mildly elevated, at 13.0 ng/mL (normal < 3.8 ng/mL). A preoperative microbiological examination of vaginal discharge was performed, which was without evidence for growth of pathogenic microorganisms. The surgically excised formation from the posterior vaginal wall was 3 × 3 cm in size with superficial mucinous secretion and propagation to the perineum (Figure 3).

**Figure 4:**
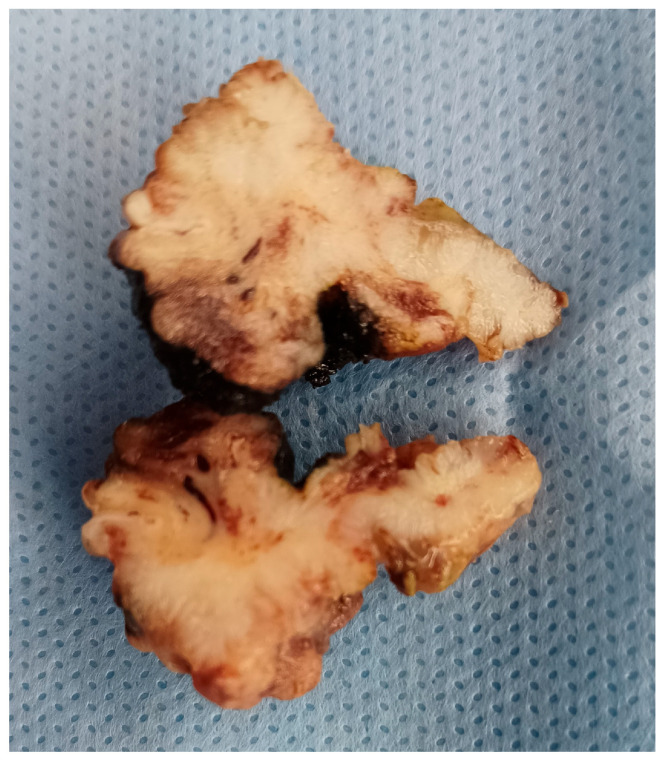
Gross examination of the surgical specimen (cut surface).

The surgical specimen was submitted for a pathological examination. Gross examination of the specimen showed a polypoid fragment measuring 3/3/1.5 cm, with an uneven surface, a soft consistency and a gelatinous appearance (Figure 4).

**Figure 5:**
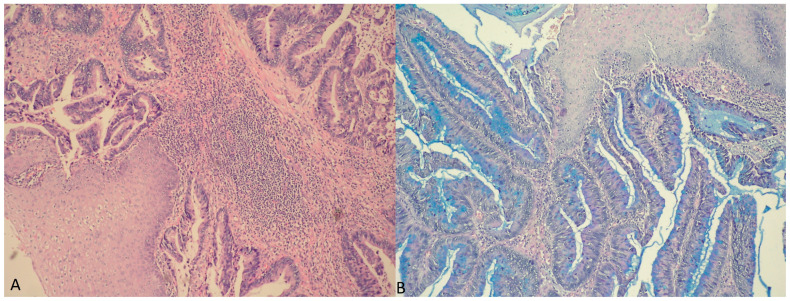
(**A**) Vaginal mucosa with infiltration of atypical glands lined by cylindrical epithelium with pseudo-stratification of cells, H&E stain, magnification ×100; (**B**) squamous epithelium (upper right corner) and mucinous adenocarcinoma intestinal type with Alcian blue positive intracellular mucus and single goblet cells; Alcian blue stain, magnification ×100.

Microscopic examination revealed a vaginal wall with a low-grade squamous intraepithelial lesion of the lining epithelium and a formation with a villous structure lined by an atypical cylindrical epithelium with pseudo-stratification of cells (Figure 5A). The latter have vacuolated cytoplasm, intracytoplasmic mucus production (Alcian blue positive), pleomorphic and hyperchromic nuclei (Figure 5B). Foci with extracellular mucin and single goblet cells were also encountered. The submucosa, the inner layer of muscularis propria and the perineural zones were infiltrated by atypical glandular structures.

**Figure 6:**
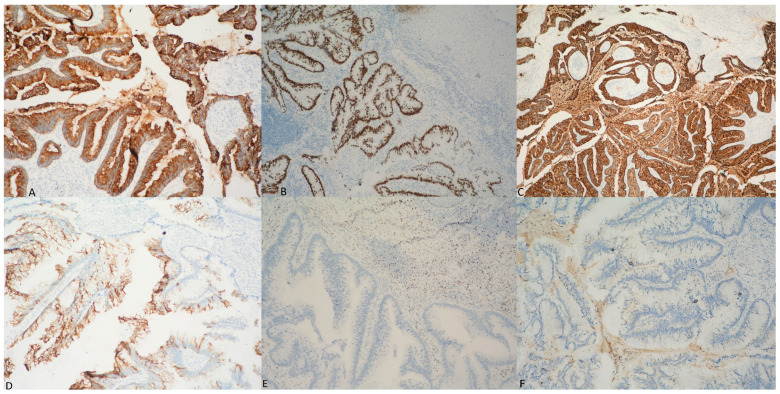
Images showing the positive immunohistochemical expression for CK7 (**A**), CDX2 (**B**), CEA (**C**), and CK20 in about 45% (**D**), in addition to the negative reaction for ER (**E**) and vimentin (**F**) in atypical glandular structures; Immunohistochemical stains, magnification ×100.

The immunohistochemical (IHC) study showed positive reaction for cytokeratin 7 (CK7) (Figure 6A), caudal type homeobox 2 (CDX2) (Figure 6B), CEA (Figure 6C), cytokeratin 20 (CK20) in about 45% (Figure 6D), Ki67 in over 50%, and negative staining for estrogen receptor (ER) (Figure 6E), vimentin (Figure 6F), and p16 antibodies. Due to suspicion of tumor emboli, D2-40 immunostaining was also performed, which ruled out lymphovascular invasion.

The positive immunoexpression for CDX2, CK20, CK7, and CEA confirmed the “intestinal differentiation” of the tumor, while the negative reactions (for ER, vimentin, and p16 antibodies) excluded metastases from other gynecological neoplasms.

Based on the pathological report, the IHC testing, and the clinical and radiological findings, the patient was diagnosed with invasive PVMAC intestinal type, arising from a villous adenoma with high-grade dysplasia. Surgical margins were tumor-free. The pathological staging of the tumor according to the TNM system was pT1NxM0 R0 PNI+, and the surgical staging according to the FIGO classification was Stage I.

**Figure 7:**
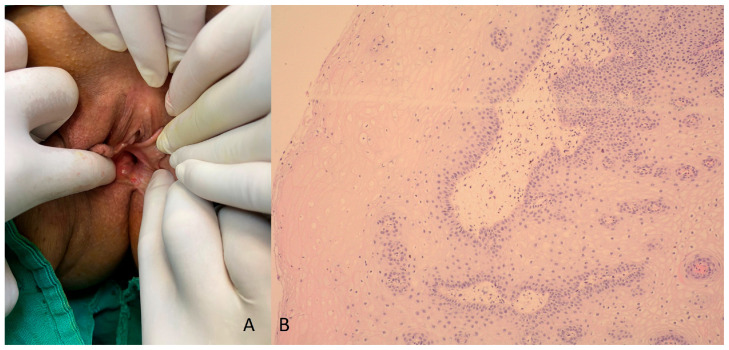
(**A**) Gynecological examination after radiotherapy. Posterior vaginal wall showed no abnormality; (**B**) biopsy from the tumor area shows squamous epithelium without infiltration—no relapse; H&E stain, magnification ×100.

**Figure 8:**
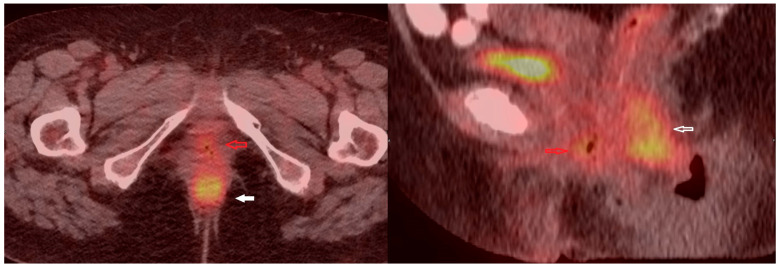
[^18^F] FDG PET/CT fused images (axial and sagittal views) showing diffuse intermediate metabolic activity in the vagina with gas inclusions (red arrow), and high metabolic activity in the anal canal due to persistence of hemorrhoidal disease (white arrow).

The patient was prescribed postoperative radiotherapy of the pelvis, with a total dose of 44 Gy. Seven months later, follow-up gynecological and histological examinations (Figure 7), as well as control PET/CT scan were performed, which did not detect disease progression (Figure 8).

## 3. Discussion

PVMAC intestinal type is a rare entity in gynecological oncology and occurs in women in a wide age range—between 36 and 86 years of age (mean—56 years old) [7,8]. Its etiology is still unclear and continues to be debated. It can arise from foci of adenosis or endometriosis with intestinal metaplasia, heterotopic intestinal tissue, cloacogenic remnants incorporated into the vaginal wall during embryonic development, as well as secondary to dysplastic enteric epithelium after surgical manipulations [5,6]. Its occurrence arising from a tubulo-villous adenoma in the vagina is extremely rare. According to the study by Broggi et al., there are only 19 cases, of which only eight are associated with neoplastic adenomatous precursors, to which he makes his contribution and adds another case associated with tubulo-villous adenoma with high-grade dysplasia [5].

The localization of primary vaginal adenocarcinomas depends on histogenesis. A lesion along the posterior border of introitus vaginae is associated with embryonic origin (from the urogenital sinus), a lateral location is associated with origin from mesonephric remnants, and those along the posterior vaginal wall are most often associated with incorporated anorectal tissue during the division process of the cloaca remnants [8,9]. The clinical manifestations of PVMAC include vaginal bleeding, foul-smelling vaginal discharge (in 65–80% of cases), and tumor masses with sizes ranging up to 70 mm in diameter [10,11,12]. In our case, the primary lesion debuted with copious mucus vaginal discharge and a small amount of irregular vaginal bleeding.

Imaging techniques such as computed tomography (CT) scan, magnetic resonance imaging (MRI), PET/CT, transvaginal and transrectal ultrasound, colonoscopy and cystoscopy might be helpful for the diagnosis of primary vaginal neoplasms [12]. CT in these cases is not an appropriate imaging method for diagnosis, as it has more limited capabilities due to the anatomical features of the organ (the small diameter and its naturally collapsed appearance). It could rather be used to evaluate disease spread or complications such as bleeding, fistulas, and intestinal obstructions [13]. Studies have shown that MRI is a very useful method for evaluating malignant vaginal tumors, as it enables the assessment of invasive growth to adjacent tissues and organs due to the contrast in signal intensity in the T2-weighted image between the hypointense vaginal wall and the hyperintense tumor lesion. The method can also be used for tumor staging [14]. In our case, MRI was not performed on the patient due to medical contraindications (knee arthroplasty). The PET/CT technique is recommended for the diagnosis of recurrent vaginal lesions and for the restaging of malignant vaginal neoplasms. This method is superior to CT, as it provides a better assessment of disease dissemination, but it is less sensitive than MRI in terms of assessing the local status [15]. In our patient, PET/CT scan was performed, which established the primary vaginal origin without evidence of lymphogenous and hematogenous spread of the carcinoma. The use of imaging techniques enables precise management of these patients both in terms of treatment plan choice as well as evaluation of the therapeutic response to a certain treatment.

Morphological examination of vaginal tumors is the gold standard for their diagnosis, and additional IHC testing validates their histogenesis. The immunoprofile of mucinous adenocarcinoma intestinal type includes a negative reaction for CK7 and a positive expression for CK20, whose constellation is more often in support of primary colorectal carcinoma, but in the case of PVMAC intestinal type there’s variable CK7 staining. On the other hand, the opposite profile (CK20 negative/CK7 positive) is suggestive of cervix, uterus, ovary, breast or lung carcinomas. Markers such as CDX2, alpha methylacyl CoA racemase (AMACR), and special AT-rich sequence-binding protein 2 (SATB2) support colonic origin. Metastases from the cervix are immunoreactive for CK7, CEA, and p16 and are negative for ER and vimentin, and disseminations from carcinoma of the endometrium and ovary are positive for paired-box gene 8 (PAX8) and negative for CK20 and CDX2. In our case, IHC examination confirmed the “intestinal differentiation” (CK20 positive, CDX2 positive, CEA positive, CK7 positive) and allowed us to exclude the gynecological metastasis possibility (negative ER, vimentin and p16 antibodies). The morphology and immunohistochemistry of PVMAC intestinal type are similar to those of carcinomas from the gastrointestinal system, which presents a serious challenge in differentiating PVMAC intestinal type and a metastasis from gastrointestinal tract, using only the immunohistochemical method. In our case, the presence of adenomatous precursors with dysplastic epithelial changes, in addition to an absence of rectal masses, suggested the possible primary vaginal origin [6,7,8,16,17,18,19,20]. Due to the limited capability of the IHC method to differentiate the PVMAC intestinal type from a gastrointestinal vaginal metastasis, the combination of clinical and morphological data, as well as instrumental and imaging studies, is decisive for making the final diagnosis of PVMAC intestinal type.

Different strategies are used for the treatment of primary vaginal adenocarcinomas, mainly radiochemotherapy and surgery, with the latter being also applicable to tumors located on the posterior vaginal wall, or in advanced-stage cases, or in cases involving recurrence or fistulization of the disease [12,14]. According to the study by Franchi et al., the surgical approach is applicable to young women (mean age 52 years old) and small size tumors (below 1.4 cm). On the other hand, women with lesions over 3.2 cm (and mean age 58 years old) are recommended radiotherapy [12].

In diagnostic and therapeutic terms, local excision with evaluation of the resection margins, partial colpectomy with resection of paravaginal soft tissues or total vaginectomy with hysterectomy and adnexectomy are also applied [12,16]. To reduce the volume of the primary tumor, combined radiochemotherapy and/or brachytherapy are considered. In advanced cases, adjuvant chemotherapy is applicable [5,12]. In our case, after the tumor extirpation (with tumor-free margins), the patient was recommended adjuvant radiotherapy.

The prognosis of primary vaginal adenocarcinomas depends on the patient’s age, tumor stage, tumor grading, localization, and the presence of regional lymphatic and distant metastases. The most important prognostic factor is lymph node status. Currently, to reduce the complications of regional lymph node dissection, sentinel lymph node dissection is performed primarily for carcinomas of the vulva and cervix. For the first time, Soergel et al. achieved sentinel lymph node dissection in a vaginal carcinoma case with the application of infrared fluorescence from indocyanine green and technetium-99 m nanocolloid [21]. Lymphatic metastatic spread of the adenocarcinoma may involve the pelvic, inguinal and supraclavicular nodes, while hematogenous spread is more common to the lungs (followed by liver and bones) and usually occurs late [22].

Due to the low incidence of PVMAC intestinal type, its prognosis remains unclear. According to Franchi et al., the median patient follow-up is 12 months [12]. Our case has been “disease free” for 16 months, up until the time of manuscript submission. The recurrence rate of adenocarcinomas of the vagina is higher than that of vaginal squamous cell carcinoma, according to Baral et al. [22].

## 4. Conclusions

Primary vaginal mucinous adenocarcinoma intestinal type is a rare pathology from the vaginal oncogynecological diseases. The small number of cases makes the disease unclear in its etiology and prognosis. Therefore, every new case with these features is significant and contributes to filling the gap in our knowledge.

## Data Availability

The original contributions presented in the study are included in the article. Further inquiries could be directed to the corresponding author.
